# Teacher-Mediated Interventions to Support Child Mental Health Following a Disaster: A Systematic Review

**DOI:** 10.1371/currents.dis.466c8c96d879e2663a1e5e274978965d

**Published:** 2015-12-08

**Authors:** Jacqueline Coombe, Lisa Mackenzie, Robyn Munro, Trevor Hazell, David Perkins, Prasuna Reddy

**Affiliations:** Centre for Rural and Remote Mental Health, School of Medicine and Public Health, Faculty of Health and Medicine, University of Newcastle, Orange, NSW, Australia; Centre for Rural and Remote Mental Health, School of Medicine and Public Health, Faculty of Health and Medicine, University of Newcastle, Orange, NSW, Australia; Priority Research Centre for Health Behaviour; School of Medicine and Public Health, Faculty of Health and Medicine, University of Newcastle, Newcastle, NSW, Australia; Hunter Medical Research Institute, Newcastle, NSW, Australia; Centre for Rural and Remote Mental Health, School of Medicine and Public Health, Faculty of Health and Medicine, University of Newcastle, Orange, NSW, Australia; Centre for Rural and Remote Mental Health, School of Medicine and Public Health, Faculty of Health and Medicine, University of Newcastle, Orange, NSW, Australia; Centre for Rural and Remote Mental Health, School of Medicine and Public Health, Faculty of Health and Medicine, University of Newcastle, Orange, NSW, Australia; School of Medicine and Public Health, Faculty of Health and Medicine, University of Newcastle, Newcastle, NSW, Australia

## Abstract

Objectives: This review sought to identify, describe and assess the effectiveness of teacher-mediated interventions that aim to support child and adolescent recovery after a natural or man-made disaster. We also aimed to assess intervention applicability to rural and remote Australian school settings.

Method: A systematic search of the academic literature was undertaken utilising six electronic databases (EBSCO, Medline, PsycINFO, Embase, ERIC and CINAHL) using terms that relate to: teacher-mediated and school-based interventions; children and adolescents; mental health and wellbeing; natural disasters and man-made disasters. This was supplemented by a grey literature search.

Results: A total of 20 articles reporting on 18 separate interventions were identified. Nine separate interventions had been evaluated using methodologically adequate research designs, with findings suggesting at least short-term improvement in student wellbeing outcomes and academic performance.

Conclusions: Although none of the identified studies reported on Australian-based interventions, international interventions could be adapted to the Australian rural and remote context using existing psychosocial programs and resources available online to Australian schools. Future research should investigate the acceptability, feasibility and effectiveness of implementing interventions modelled on the identified studies in Australian schools settings.

## Background

**The impact of disaster in Australia**

Between January 2010 and January 2015, Australia experienced 31 natural disasters including cyclones, floods, storms, tornados and bushfires^1^. While all Australians are impacted by natural disasters, those in rural and remote communities are at increased risk of experiencing one or more natural disasters in their lifetimes^2^. A national strategy for disaster resilience has been developed to guide the planning and implementation of prevention, preparation and recovery efforts^3^. As understanding of the psychological impact of natural disasters has developed, so to have the range of responses addressing these impacts^4^. However, the implementation of responses (including psychological support responses) to sudden onset disasters is associated with different challenges compared to responses to gradual onset disasters (e.g. chronic weather events) or man-made disasters.

For the purpose of this article, natural disasters refer to sudden onset of significant environmental events, including bushfires, flood and cyclones. Man-made disaster refers to war or terrorist-related activities, rather than human induced environmental disasters such as oil spills or environmental degradation.

**Child and adolescent mental health following a disaster**

The trauma that children and adolescents may experience as a result of disasters is widely explored^5^^,^ 6 and the resultant emotional and behavioural adjustments that can be triggered by a traumatic experience well known. Increased rates of depression and anxiety among children following a natural disaster have been reported^7^ with some evidence of symptoms persisting over time^8^. Additionally, research conducted in Australian schools in the aftermath of bushfires identified elevated rates of post-traumatic stress disorder (PTSD) among students^9^; a finding comparable to global PTSD rates among children who have experienced a range of other natural disasters 10 ^,^ 11 ^,^ 12. Exposure to man-made disasters, such as war and terrorism, may also elicit PTSD symptoms among children and adolescents.^13^

Children who are diagnosed with PTSD often report a decrease in post-disaster quality of life^14^ These responses to trauma can emerge as negative classroom behaviours, including poorer grades, difficulty concentrating and disruptive behaviours^15^^,^ 16. There is growing recognition of the need for supportive interventions targeting child and adolescent mental health and other behavioural outcomes after a disaster 17 ^,^ 18.

**The rationale for school-based, teacher-mediated interventions in Australia**

It has been argued that teachers are ideally placed to deliver post-disaster mental health programs and support to their students in the school setting^19^ for a number of reasons. Schools play a key role in the aftermath of a disaster^20^, and are often used as relief sites, community information hubs or supply depots^21^. Additionally, schools are typically one of the first organisations to resume operations after a disaster, and can provide students with a sense of returning to normality^22^. Through their existing network of teachers, parents, peers and students, schools provide a non-stigmatising setting for seeking and receiving psychological support^19^^,^ 23. This has particular relevance in rural and remote areas of Australia, where access to health resources are traditionally limited.^1^^,^ 19

In Australia, the school setting is increasingly used to deliver support programs for students who experience trauma associated with family breakdown or the loss of a loved one. The Seasons for Growth program is an example of this^24^. There have also been increasing efforts by schools to implement programs to support and address other aspects of youth mental health such as youth depression and drug and alcohol use, with programs including MindMatters^25^ and KidsMatter^26^. Although disaster education is included in the Australian curriculum, a recent study conducted by Dufty^27^ highlighted further opportunity for the inclusion of disaster resilience strategies.^26^

**Objectives**

The primary aim of this review was to identify describe, and determine the effectiveness of, school-based teacher-mediated interventions developed to support child and adolescent mental health and wellbeing following either a natural disaster or a man-made disaster. The secondary aim was to identify interventions conducted in or relevant to the Australian rural and remote schools.

## Method

**Search strategy**

This review was conducted in accordance with the PRISMA guidelines for systematic reviews.^28^

**Eligibility criteria**

All literature identified by our search as reporting on school-based, teacher-mediated interventions to support child and adolescent recovery after a disaster was included in the review. Studies were included if they clearly described that the intervention was delivered by the classroom teacher to their students or if they described an intervention for teachers, and/or reported the outcomes of these interventions on students or the teachers. Interventions facilitated by therapists/counsellors or other health professionals in partnership with teachers were excluded, as these were not considered to be relevant to the Australian rural or remote school context. All study designs were included as long as they met the above eligibility criteria. No restrictions on the start date of the search period were imposed; the end date of the search period was July 2013, which has been subsequently updated to January 2015. The search was restricted to English language and peer-reviewed journal articles. No further search restrictions were applied.

**Information sources**

Six electronic databases were searched: EBSCO, Medline, PsycINFO, Embase, ERIC and CINAHL. These databases were chosen due to their target audience; health, psychology and education.

**Search strategy**

Each database was searched using terms that relate to: teacher-mediated and school-based interventions; children and adolescents; mental health and wellbeing; natural disasters and man-made disasters. For example, the specific search terms used in the EBSCO database were: (teacher mediated OR teacher led OR teacher based OR school based OR school led OR community based OR social support) AND (child OR children OR adolescent OR child, preschool OR teenagers) AND (grief OR anxiety OR stress, psychological OR stress disorders, post-traumatic OR depression OR trauma OR coping OR mental health OR wellbeing OR social capital OR resilience OR connectedness OR adaption, psychological) AND (disasters OR natural disaster OR bushfire OR wildfire OR floods OR cyclonic storms OR cyclone OR earthquake OR tsunami OR man-made disaster OR terrorism OR terrorist attack OR war OR civil war).

**Study selection**

All identified articles were added to an Endnote library. Duplicates were removed using the Endnote “remove duplicates” function, and through manual scanning. Based on titles, articles returned in the search that were clearly not relevant were excluded. The remaining articles were assessed based on abstracts and key words, and excluded if they did not meet the inclusion criteria. If no abstract was available full text articles were retrieved and reviewed to assess relevance. If there was uncertainty regarding article inclusion, a co-author was consulted until agreement was reached.

**Data items**

Data extraction tables were used to record characteristics of included studies. Studies were grouped as being relevant to either 1) natural disaster events or 2) man-made disaster events. Additional data extraction items and grouping/classifications applied to studies included: a) Study design;b) Disaster of interest; c) Participant characteristics; d) Intervention characteristics; e) Teaching training characteristics; and f) Results. In order to make a judgement about the effectiveness of the identified interventions, articles were then categorised based on whether the study design conformed to Effective Practice and Organisation of Care (EPOC) guidelines^29^. For studies that met EPOC research design criteria, information on measures, length of follow-up and direction and significance of study outcomes were extracted. Due to the heterogeneity of outcome measures across studies, the findings were not deemed to be suitable for synthesis via meta-analysis.

**Risk of bias**

Risk of bias was assessed for each study meeting the EPOC criteria, utilising the suggested risk of bias criteria for EPOC reviews^29^. Two reviewers assessed each article separately, before comparing their results. Consensus was reached for each study.

**Grey literature search**

Additional to the database search, a series of Google searches were conducted during July 2013, and subsequently updated in January 2015 to explore resources currently available to teachers online. Google searches used simplified combinations of the electronic database search terms, with a focus on Australia; for example ‘disaster resources Australia teachers’. While the limitations of this grey literature search strategy are acknowledged^30^, the search aimed to develop an understanding of the types of disaster resources that are accessible to Australian teachers, and not to provide an exhaustive resource. The resources were classified by a) organisation; b) specific type of disaster; c) description of resource; and d) web link to resource.

## Results

**Study selection**

Figure 1 describes the process of article identification, screening, and full text article retrieval and assessment for inclusion. A total of 1652 articles were retrieved, of which 20 articles reporting on 18 separate interventions were deemed relevant to this review.

**Process of selection of papers for review:**
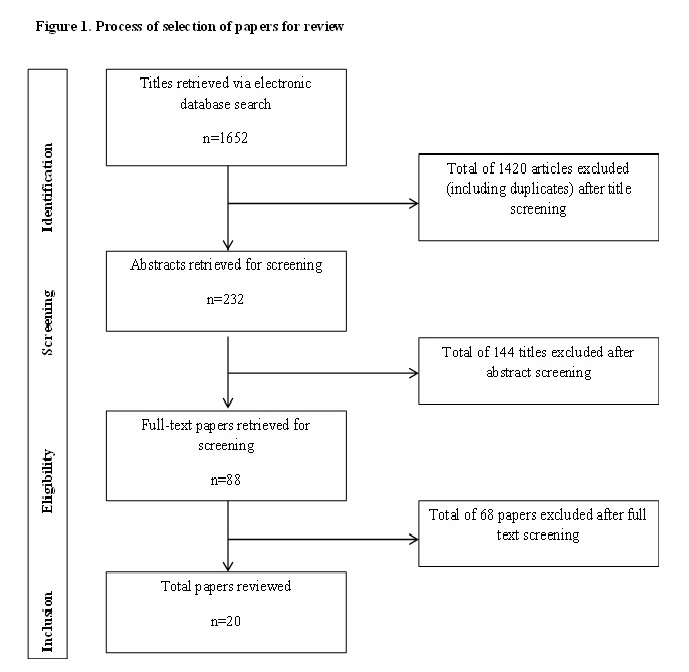

**Included peer-reviewed papers**

Of the 20 articles, nine described seven interventions following a natural disaster and 11 articles described interventions in response to man-made disasters. The 18 interventions broadly fell into one of two categories: They either a) reported on the implementation of the intervention in the classroom and its outcomes for students (n = 12), or b) reported on the process of training teachers (n= 6). None of the interventions identified were conducted in Australia. Each intervention (categorised as being a response to either a natural or man-made disaster) is outlined in Table 1.

Table 1 - Identified Interventions

**Studies with EPOC designs**

Table 1 indicates that nine of the 18 studies were deemed to meet EPOC design criteria, and Table 2 summarises the findings of these nine studies. Three studies related to natural disasters (2 classroom-based, 1 teacher training) and 6 studies related to man-made disasters (5 classroom-based, 1 teacher training).

Table 2 - EPOC Design Studies

**Study characteristics**

Of the three studies focusing on natural disasters that met EPOC research design criteria, one focused on outcomes for teachers with the study^31^ reporting an intervention effect on teacher’s professional self-efficacy and a range of other outcomes (see Table 2). The two classroom based interventions reported on general child-functioning outcomes (including academic performance and social behaviour) described intervention effects^19^^,^ 32 ^,^ 33. Although there was some evidence of short-term intervention effects on mental health outcomes (including PTSD symptomology) in these studies at 3-months follow-up^19^^,^ 33, no effects were found 3-years post-intervention 32 (see Table 2).

Five out of the six studies focusing on man-made disasters that met EPOC research design criteria reported data on child and adolescent mental health outcomes. As seen in Table 2, all studies reported reductions in PTSD symptomology 34 ^,^ 35 ^,^ 36 ^,^ 37, and a range of other mental health and functional/behavioural outcomes. One study indicated that three students in the intervention group actually developed PTSD during the course of the intervention^36^, however this outcome was not attributed to the intervention. The only study reporting any contraindication for an intervention was Wolmer et al. 37, where the grief outcomes worsened post-intervention (see Table 2).

**Risk of bias**

Risk of bias was assessed using EPOC guidelines^29^ More than half of the EPOC studies were categorised as high or unclear risk in the domains of sequence generation and allocation concealment, primarily due to their study design. Additionally, in the domain of ‘other sources of bias’ unclear risk was given to all studies as they did not address other possible areas of bias and assessors could not adequately judge if bias occurred. For example: it was unclear if students from control and intervention groups interacted about the program while it was underway, or if teachers in the control who received the training but were told not to implement it, either inadvertently or consciously delivered the training to students. All studies were scored either low or unclear risk on the remainder of the risk of bias domains (see risk of bias table 3).

Table 3 - Risk of Bias

**Resources available to Australian schools**

Table 4 details the disaster resources provided by government and non-government organisations in Australia and New Zealand that were identified through the grey literature search. The resources fall into three categories: 1) information and fact sheets for teachers; 2) lesson plans and interactive resources to be implemented in the classroom; and 3) teacher training. Some resources are disaster-specific (e.g. bushfires), while others are more general. Almost all resources focused on natural disasters. Please see Supplementary Table 1 for further detail about these resources, including URLs. The grey literature search did not return any of the included studies in this review.

Table 4 - Grey Literature

## Discussion

We identified seven school-based teacher-mediated interventions that were developed to support child and adolescent mental health and wellbeing following a natural disaster, and 11 following a man-made disaster. Eight interventions were evaluated using methodologically rigorous designs, reporting significant short-term intervention effects on some student outcomes (including reduced psychological symptomatology and increased academic performance) and teacher outcomes (including increased personal and professional self-efficacy). Although none of the identified interventions were conducted in Australia, there is potential to adapt effective international models, drawing upon nationally relevant disaster recovery resources that are freely available online.

**Description of the identified interventions**

The greatest disparity evident between interventions responding to natural versus man-made was commencement time. Natural disaster interventions commenced immediately to 15 months after the disaster, while interventions targeted at man-made disasters were implemented across a broader timeframe. Differences between natural and man-made disaster interventions were also evident in their reported outcomes. Natural disaster targeted interventions more often reported behavioural changes, while man-made disaster interventions reported clinical changes and behavioural changes. Behavioural changes may be most relevant to teachers if they assist school performance and do not require clinical knowledge.

Interventions were categorised according to their intervention type as either classroom based or teacher training with there being slight differences between these two categories (see Table 1). Classroom based interventions involved the classroom teacher delivering class sessions lasting between 45 minutes to two hours, which occurred on a regular basis over a number of weeks. These sessions focused on building resilience and coping strategies and included play therapy, activities to develop emotional awareness, conflict resolution, drama games, and cooperative learning, the timing of the implementation of the intervention relative to disaster onset and the duration of the interventions varied. Comparatively, teacher training interventions reported on the process of training teachers in disaster preparedness and response and provided the tools and resources to use personally or in the classroom. These were most often facilitated by mental health workers. A number of studies reported using the ‘train the trainer’ technique, which was often used to reach a large number of schools and students. All teacher-training articles focused on the goal of providing the teacher with the tools and resources to support their own recovery and to implement a mental health program in their classroom without the need for external services running the ongoing program.

**Effectiveness of the identified interventions**

Of the interventions that were evaluated using an EPOC endorsed rigorous research design, positive findings suggest that the identified interventions may be effective for improving teacher self-efficacy and student academic, mental health and wellbeing outcomes, at least in the short term. Based on heterogeneity of intervention components, outcomes measures and follow-up periods, we were unable to determine which particular interventions characteristics were associated with which outcomes. Further methodologically rigorous research assessing similar outcomes is required before conclusions can be drawn about intervention effectiveness.

**Relevance of the identified interventions to the Australian context**

Despite none of the interventions being conducted in Australia, a number of the interventions reported here were based on a particular disaster-recovery strategy (for example ERASE-Stress 31 ^,^ 33 ^,^ 34 ^,^ 36), which was applied to different cultural contexts and disasters. Potentially these strategies could also be adapted to the Australian context. There are a number of online resources available to Australian teachers from reputable sources, including a number of Australian government departments and Non-Government Organisations. The recent redevelopment of MindMatters for use in secondary schools is one such example. MindMatters provides online resources and guidance for schools in developing and implementing mental health strategies applicable to their individual school environments^38^. Online resources often focus on developing students’ psychosocial skills, such as increasing resilience. Given that most of the interventions identified in this review similarly aimed to develop resilience, general online resources with a focus on building resilience and wellbeing could potentially be used by teachers post-disaster in the absence of an Australian specific intervention. As highlighted in the introduction, programs such as these are already used in schools to address other aspects of mental health and wellbeing; post-disaster implementation may be feasible.^31^^,^ 33 ^,^ 34 ^,^ 36 ^,^ 37

Consultation with teachers to identify their needs (both personal and professional) after a disaster may be the most logical next steps for this area of investigation. Consultation with Aboriginal communities would also be a crucial aspect of any intervention development. Further investigation into resources available to Australian teachers, particularly the usefulness of these resources and their ability to be implemented into the classroom may also prove beneficial. A recent study has found that approximately 50% of the teachers sampled felt that they do not have enough time to meet the mental health needs of their students^39^. Thus, consultation may help ensure that teachers would not be over-burdened with complex intervention strategies that require high level mental health training, and allow greater integration into the classroom environment.

**Review limitations**

Publication bias may be evident as none of the included studies reported significantly negative findings. Further, due to the difficulty of implementing and evaluating interventions in a post-disaster environment there may be interventions with successful and positive outcomes that have not been reported in the peer-reviewed literature. Many non-relevant studies were also identified through the search procedures employed. A preoccupation with PTSD was evident and the authors acknowledge the need for broader strategies addressing issues above and beyond PTSD are essential. As the inclusion criteria was limited to interventions that describe teachers delivering the support program interventions may have missed that have other school staff delivering the program. It is worth noting that if the school has resources, including other staff such as chaplains or school counsellors that can be trained to deliver interventions may be an option for some schools.

While our search criteria sought to capture teacher mediated class room based interventions we acknowledge that interventions may have been missed using such a strict search strategy. Interventions such as the Classroom-Based Intervention (CBI)^40^, which highly involve teachers and schools during the development and delivery but used CBI trained guidance counsellors to deliver the classroom sessions with the support of teachers, have been missed using this search strategy. These types of studies are relevant to the field and provide evidence and support for classroom based disaster recovery programs.

We have defined man-made disaster as war or terrorist-related activities, rather than human induced environmental disasters such as oil spills or environmental degradation. Although no interventions were identified which specifically addressed human-induced environmental disasters, it may be possible that our selection of search terms may have inadvertently excluded any literature discussing these types of disaster interventions. Additionally, drought was not included our search terms as it is not currently considered a natural disaster in Australia under the Australian Government’s Natural Disaster Relief and Recovery Arrangements^41^. Further, responses to chronic weather events, such as drought, differ from responses to sudden onset natural disasters^42^.

The grey literature search identified resources related to resilience and wellbeing from organisations such as Beyond Blue and KidsMatter. While these resources are readily available to schools, as they were not disaster recovery specific they were not reported in this review. However, as resilience building and increasing wellbeing are often the focus of disaster recovery interventions, programs such as these could be included in future research exploring resources available to teachers post-disaster.

## Conclusion

Teacher-mediated interventions may have the potential to improve child and adolescent mental health after a disaster, although findings are inconclusive. International studies provide a useful guide for the development, implementation and evaluation of teacher-mediated disaster-response mental health programs for Australian schools. Further investigation is necessary before conclusions can be drawn about the applicability of these identified international interventions to the Australian rural and remote context. In the absence of recommendations for evidence-based teacher-mediated disaster interventions, resources aimed at increasing resilience and wellbeing may be useful for teachers attempting to manage disaster recovery in their classrooms, particularly in rural and remote locations where provision of services by mental health professionals is lacking.

## Competing Interest

The authors have declared that no competing interests exist.

## Appendices

Supplementary Table 1. Disaster Resources Summary and URL

PRISMA Checklist
